# A study on effects of and stance over tuition fees

**DOI:** 10.3205/zma001005

**Published:** 2016-02-15

**Authors:** Yassin Karay, Jan Matthes

**Affiliations:** 1 University of Cologne, Medical Faculty, Dean's Office for Student Affairs, Cologne, Germany; 2 University of Cologne, Department of Pharmacology, Cologne, Germany

**Keywords:** tuition fee, student dropouts, course of studies

## Abstract

**Aim:** Regarding tuition fees (that in Germany already have been abrogated) putative drawbacks like prolonged study duration have been suspected while benefits are not clearly proven. We investigated whether tuition fees (500 Euro per semester) affected the course of studies of Cologne medical students and asked for students’ stance over tuition fees.

**Methods: **Of 1,324 students we analyzed the rate of those passing their first medical exam (“Physikum”) within minimum time and students’ discontinuation rate, respectively. Regression analysis tested for putative influences of tuition fees and demographic factors. In an additional online survey 400 students answered questions regarding the load by and their stance over tuition fees.

**Results: **We find that fees did not affect rate of Cologne students passing their first medical exam within minimum time or students’ discontinuation rate. According to the online survey, at times of tuition fees significantly more students did not attend courses as scheduled. Time spent on earning money was significantly increased. 51% of students who had to pay tuition fees and 71% of those who never had to stated tuition fees to be not justified. More than two thirds of students did not recognize any lasting benefit from tuition fees.

**Conclusion: **Tuition fees did not affect discontinuation rate or study duration of Cologne medical students. However, they obviously influenced the study course due to an increased need to pursue a sideline. Cologne medical students rather refused tuition fees and did not recognize their advantages in terms of enhanced quality of studies.

## Zusammenfassung

**Zielsetzung: **Durch die in Deutschland zurzeit wieder abgeschafften Studiengebühren wurden Nachteile wie z.B. eine verlängerte Studiendauer befürchtet, während der Nachweis eines Nutzens bislang aussteht. Wir haben den Einfluss von Gebühren in Höhe von € 500 pro Semester auf den Studienverlauf Kölner Medizinstudierender sowie deren Einstellung gegenüber diesen Abgaben untersucht.

**Methodik: **Für 1.324 Studierende, die im vorklinischen Studienabschnitt durchweg, zeitweise oder nie Studiengebühren zu zahlen hatten, wurde die Einhaltung der Mindeststudienzeit und die Studienabbrecherquote bis zum ersten Abschnitt der ärztlichen Prüfung analysiert. Mithilfe einer Regressionsanalyse wurden etwaige Effekte durch Studiengebühren und demografische Faktoren untersucht. Zusätzlich beantworteten 400 Studierende in einer online-Befragung Fragen zu ihrer Belastung durch und ihrer Einstellung zu Studiengebühren.

**Ergebnisse: **Studiengebühren hatten keinen erkennbaren Einfluss auf Studiendauer oder Abbrecherquote. Laut Angaben in der online-Befragung wichen zuzeiten der Studiengebühren signifikant mehr Studierende vom vorgeschlagenen Stundenplan ab und sie verwendeten signifikant mehr Zeit darauf, nebenher Geld zu verdienen. 51% der Studierenden, die Gebühren gezahlt hatten und sogar 71% derer, die dies nie mussten, fanden Studiengebühren nicht oder eher nicht gerechtfertigt. Mehr als zwei Drittel der Studierenden konnten keine anhaltende Verbesserung durch Studiengebühren erkennen.

**Schlussfolgerung: **Studiengebühren hatten an der Medizinischen Fakultät zu Köln keinen Einfluss auf Abbrecherquote und Studiendauer. Sie veränderten aber offenbar das Studierverhalten durch die vermehrte Notwendigkeit, neben dem Studium Geld zu verdienen. Die Kölner Medizinstudierenden sahen die Studiengebühren mehrheitlich kritisch und nahmen keinen nachhaltigen Nutzen im Sinne einer Verbesserung der Ausbildungsqualität wahr.

## 1. Introduction

### 1.1. Background

In many but not all countries, students have to pay fees for their studies. While for example in the U.S.A. tuition fees are an inherent part of the educational system this is not the case in many European countries [1], [2]. In Germany, tuition fees were never demanded nationwide and have been of rather moderate amount (1,000 € per year maximum). Nonetheless, they repeatedly have been a matter of controversy. While those who advocate tuition fees argue that teaching quality would be improved by raising tuitions, their opponents fear that (potential) students – particularly those with a weak socio-economic background – would be kept from entering higher education at all [3], [4], [5]. North Rhine-Westphalia was one of the German federal states where tuition fees have temporarily been mandatory. Students of the University of Cologne had to pay 1,000 € per year (500 € per semester) from 2006 until the federal state Government abrogated these fees in 2011. It is still unclear whether tuition fees had had an impact on students’ study course. For example, it has been suspected that studies would be prolonged or dropout rates increased since students are significantly burdened by earning money to pay tuitions [6], [7].

#### 1.2. Aim of the study

We wanted to investigate whether tuition fees of rather moderate amount had an effect on course and success of medical studies. Furthermore, we conducted a prospective online survey to ask medical students about their stances over tuition fees and whether they have been burdened by this duty. In addition we wanted to test for

1. putative effects of stance and/or (perceived) burden on students’ study course and
2. putative effects of tuition fees on students’ study course and stance over these fees.

## 2. Methods

### 2.1. Setting

In accordance with the German Medical Licensure Act (“Approbationsordnung”) the reformed medical curriculum in Cologne is divided into three parts and takes at least six years (12 semesters) in total, similar to regular medical curricula [8]. The first section is a two-year preclinical part, the second section is a three-year clinical part and the last section is a one-year internship (“Praktisches Jahr”) in medicine, surgery and a discipline of choice.

#### 2.2. Retrospective analysis of course of studies and dropout rate

Assessment results and study progress of medicals students at the University of Cologne are documented and administrated in the local campus management system. By analyzing data from subsequent cohorts, we calculated the rate of students finishing their preclinical studies in the minimum time period, and the number of students per cohort leaving the university (dropout rate) during their preclinical studies [9]. Using a multivariate regression analysis, we tested for the influence of the amount of tuition fees paid, gender, age, nationality, and the grade of general qualification for university entrance (“Abitur”) on rates of students passing in minimum time or leaving university early, respectively.

#### 2.3. Online survey

By e-mail we invited all medical students enrolled at our Medical Faculty at the time of our study to attend a survey that was offered online via the e-learning platform ILIAS. The self-developed items aimed at students’ views on tuition fees and their impact on students’ course of studies [see attachment ]. Answers had to been given using a five-point Likert scale (from “totally agree“ to “totally disagree“) or simply by affirmation (“yes”) or rejection (“no”). The survey was available online from December 17^th^ 2013 to February 20^th^ 2014. Some items were only presented to those students who stated that they had paid tuition fees in the past. A translated version of the applied questions is available as an online supplemental. Survey data were analysed descriptively or in comparison depending on whether only students who had to pay tuition fees or all students should answer the particular questions.

#### 2.4. Sample selection

Data of eight cohorts (enrolment from winter term 2008/2009 until summer term 2012) were included into the analysis of course of studies, covering 1,324 students in total. Three cohorts paid 500 € per semester throughout their preclinical studies (four terms, i.e. 2,000 € in total), three cohorts had paid tuition fees for three, two or one term, respectively (i.e. 1,500, 1,000 or 500 € in total, respectively). Two cohorts never had to pay tuition fees during their studies.

The invitation to attend the online survey was sent via e-mail to all students regularly enrolled at our Medical Faculty at the time of our study (N=2,285 in total).

#### 2.5. Variables

Regarding course of studies and dropout rate we only analyzed the “preclinical” part of medical studies (minimum of four terms, completed with the first medical exam “Phsyikum”) since dropout rates are negligible during the clinical part of medical studies. As endpoints we defined “kept minimum time of four terms to finish preclinical studies“ (no=0, yes=1) and “left university during first two years of study” (no=0, yes=1). Based on Chi^2^ tests, Spearman correlations and a binary regression analysis we investigated how tuition fees, gender, age, nationality, and grade of general qualification for university entrance might have influenced course of study and dropout rates, respectively.

#### 2.6. Data privacy and ethics

All data were collected and analyzed anonymously. The Ethics Committee of the University of Cologne raised no concerns regarding this study or the publication of the results (reference No. 15-377).

## 3. Results

### 3.1. Retrospective analysis of course of studies and study discontinuation rate

**Figure 1:**
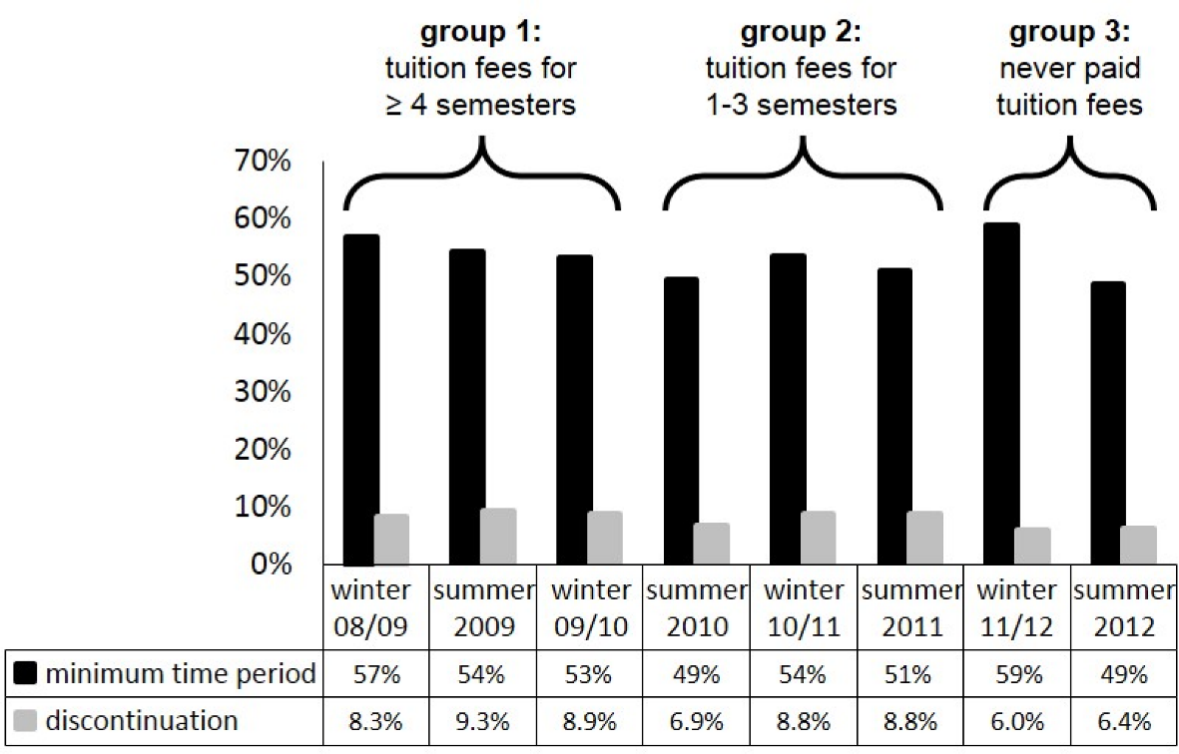
Fraction of students keeping standard study period (black columns) and fraction of students leaving university during the first two years of their studies (grey columns) during the study period. There was no association to amount of tuition fees paid. Winter: winter term; summer: summer term.

The rate of students needing only the minimum study period in the preclinical phase did not differ significantly between the observed cohorts (Chi²=5.95, df=7, p=0.55) (see figure 1 (Fig. 1)). According to a correlation analysis there seems to be no link between keeping minimum study period and the level of tuition fees (r=0.01, p=0.72). Furthermore, categorization of the eight cohorts into three groups (group 1: ≥ 2,000 € tuition fees; group 2: 500-1,500 € tuition fees; group 3: no tuition fees) revealed no difference between groups regarding the standard study period in the preclinical phase (Chi²=1.2, df=2, p=0.55). 

We also compared the dropout rates within the first four semesters and found no differences for the eight cohorts (Chi²=2.67, df=7, p=0.91) or the three groups mentioned above (Chi²=2.0, df=2, p=0.37), respectively (see figure 1 (Fig. 1)). Correlations between dropout rates and tuition fees were not significant (r=0.03, p=0.24).

Using a binary logistic regression, we analyzed the strength and direction of the influence of independent variables on the standard study period and dropout rate. Lower age, better school grades and German nationality were positive predictors regarding the standard study period throughout (see table 1 (Tab. 1)). We found a significant effect of tuition fees in the subgroup of students with excellent grades. The more tuition fees they had paid, the more likely they were to study within minimum study period. In the subpopulation of foreign students, women passed in minimum time significantly more often than male students. Except for the subgroups "foreign students" and "excellent school grades" age was predictive for dropout (see table 2 (Tab. 2)). Gender, nationality, school grades and tuition fees showed no effect on dropout rates.

#### 3.2. Survey data on course of studies and stance over tuition fees

From the online survey we obtained 400 observations (response rate of 18% given a total number of 2,285 medical students enrolled at the University of Cologne at that time). Regarding gender, nationality or age there was no difference between our sample and the entire population. Therefore, we consider our sample to be representative regarding these attributes. 

The online survey revealed that the percentage of students with parents having a university degree and students with parents without university degree was similar for tuition fee payers and non-payers (72%). When asked how tuition fees were paid, 40% of the respondents stated to have jobbed while studying and 17% to have taken out a loan to pay tuition fees. At times of tuition fees, 74% of the jobbers stated to have worked more than 11 hours per week, while after abrogation of tuition fees this fraction dropped to only 46%. Nearly half of the payers (44%) responded that working was one of the reasons for extending the standard study period. 

**Figure 2:**
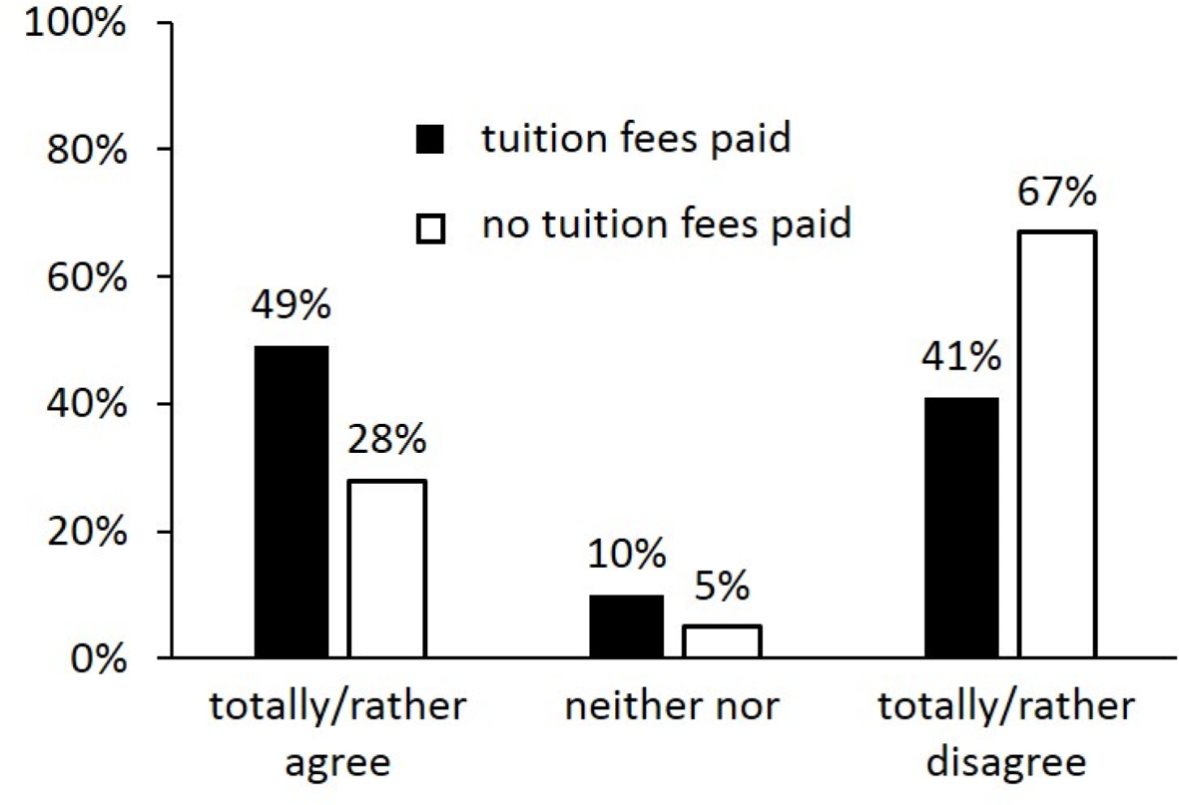
Distribution of answers students gave to the question whether they had preponed or delayed courses during their studies.

Less than a third of non-payers (28%) but approximately half of payers (49%) indicated to have switched (i.e. delayed or preponed) courses for at least one semester (Chi²=40.23; df=4; p<0.01) (see figure 2 (Fig. 2)). Obviously, the tuition payers could catch up: according to their own statement, 48% of the payers and 47% of the non-payers completed successfully the preclinical phase in minimum study period, which fits with the results of our retrospective analysis.

#### 3.3. Survey data on course of studies and stance over tuition fees

**Figure 3:**
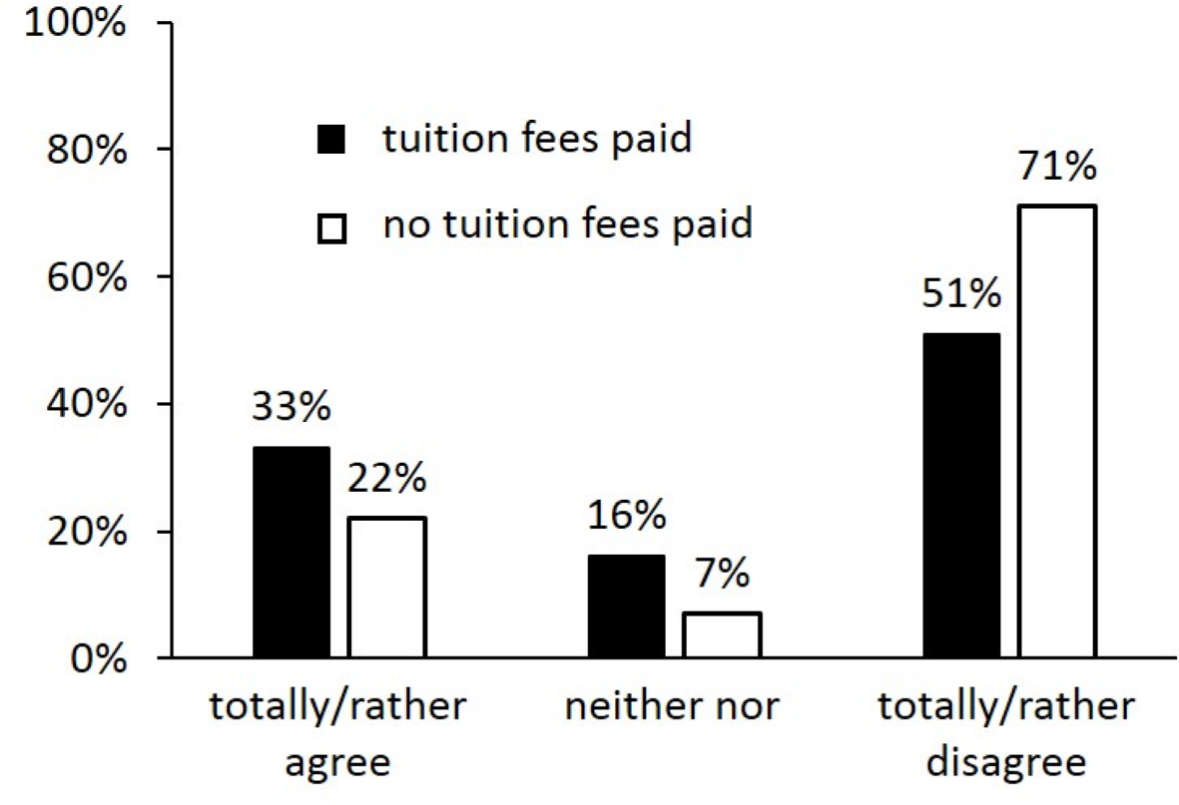
Distribution of answers students gave to the question whether “raising tuition fees is justified”.

Just over half of the payers (51%) stated that tuition fees in medical studies are not or rather not justified (see figure 3 (Fig. 3)). This result was even stronger for non-payers with 71% (Chi²=14.72; df=4; p<0.01).

Three quarters of the payers disliked the reintroduction of tuition fees of about 500 € per semester. The negative attitude towards the reintroduction of tuition fees was even stronger for non-payers (88%; Chi²=11,456; df=4; p<0,05). Interestingly, 70% of the payers stated that the quality of the studies did not improve considerably at times tuition fees were raised. Only 29% of non-payers and 27% of payers thought that the study environment had effectively improved by tuition fees.

## 4. Discussion

In this study we find that tuition fees of 1,000 € per year did not affect proportion of Cologne medical students studying in minimum time nor fraction of students quitting their studies early. There was a moderate but statistically significant effect on how students organize their schedules (i.e. preponing or delaying courses). At times tuition fees were raised, students spent more time on earning money. The majority of students attending our survey had a rather negative attitude towards tuition fees. Interestingly, this was more common among students who did not have to pay tuition fees at all.

### 4.1. Socio-economic background

In public and media it is often claimed that tuition fees would keep graduates with a low socio-economic background from enrolling in higher education. In fact, this seems not to be the case in Germany [10], [11], but: [12]. Though Quast et al. found that willingness to enroll was lower among people with a lower socio-economic background, this was the case both in German states with and without tuition fees, respectively [4]. In agreement with this, the fraction of students whose parents have a university degree was 72% in our study independent of whether tuition fees were demanded at time of enrolment or not. It is important to note that students at the University of Cologne had to pay rather moderate tuition fees of 1,000 € per year (nota bene the maximum value raised at public universities in Germany) [12]. Accordingly, it has been argued that the quite low fraction of students with a low socio-economic background in Germany is rather due to social factors in general than to tuition fees in particular [13]. 

Advocates of tuition fees often point to the quite high fraction of enrolled students with a low socio-economic background in the U.S.A. despite tuition fees being significantly higher than in Germany [14]. However, when looking at tier 1 colleges (i.e. Doctoral/Research Universities) - that are most similar to German universities - the fraction of students from families with low income is rather low as well [15]. Of note, there are data suggesting that public share of expenditure (e.g. to compensate for tuition fees) helps to limit negative enrolment effects that are due to liquidity constraints [16], [17]. On the other hand Kane found that lowering tuition fees in general had a greater effect on the decision to enroll than had targeted support aiming at reducing financial burden of needy people [18], [19]. This might be explained by a discouraging effect of tuition fees per se that already prevents needy people from looking for financial support at all. In Ontario (Canada) tuition fees doubled from 1997 to 2000 up to about 12,000 CAD per year (ca. 9,100 €) and in the same time the proportion of medical students from families with low income decreased from 23% to 15% [20]. Merani et al. found that across Canada, higher tuition fees was a major factor associated with higher debt at the time of graduation [21]. They found that nearly 90% of medical students expected to graduate with debt. According to our survey, only 17% of Cologne medical students took out a loan to pay their tuition fees of 1,000 € annually. In the UK, an increase of tuition fees by 8,000 € resulted in a significant negative effect on enrolment of older students while students at an age about 18 years where not affected [2]. Taken together, depending on their amount tuition fees might have an impact on particular subpopulations (e.g. those from low-income families or older students).

#### 4.2. Time spent on earning money

Another objection frequently raised is that students have to spend additional time on earning money to cover tuition fees and this has negative effects on successful graduation. Eventually, this might lead to a delay or even an abortion of their studies. In our study, hours worked per week indeed increased at time of tuition fees but this did neither affect duration of preclinical studies nor dropout rates. This might be explained by the finding that moderate tuition fees rather reduce leisure time than intensity of studies [7]. Assuming a lower flexibility of elderly students due to private obligations (e.g. own family) this particular subpopulation might be more vulnerable to increased expenditure. However, in our study we found no age-dependent effect of tuition fees on duration of studies or dropout rate, respectively. Though one might argue that tuition fees in Cologne have been rather low other studies have shown that public share aiming at reducing financial burden of students was accompanied by even prolonged duration of studies and lower performance of students [16], [22]. One can speculate, that recognizing medical studies as a course of studies with very high prestige might have prompted students to successfully finish “at all costs”. This might explain the strong willingness to pursue a sideline or to deviate from the suggested schedule, by this accepting that study duration might increase.

#### 4.3. Quality of medical studies

Advocates of tuition fees argue that tuition fees would help to enhance quality of studies. To the authors’ knowledge there is no clear-cut evidence for this. In fact one should expect that the effect of tuition fees is inherently linked to the reason why these fees are demanded. For example, tuition fees might be used to compensate for increased expenditure necessary to maintain the status quo. Thus, one should not expect that tuition fees are inevitably associated with increased quality of studies. In Canada student-to-staff ratio as a frequently used measure of study quality rose by 20% although tuition fees continuously increased [2]. In Germany, tuition fees were mainly levied to increase the quality of studies. Nonetheless, for German students the application of tuition fees has been used as both an argument for and against tuition, respectively [23]. In Cologne, a local committee mainly consisting of students decided how tuition fees were spent. The more surprising is that in our study students mainly did not see improvements attributable to tuition fees. To illustrate our surprise we give two examples: in Cologne, a four-storied building comprising among other things a high-class skills lab [http://kiss.uni-koeln.de], the office of the students’ council, and recreation rooms for students was largely funded by tuition fees. A one-week practical course for 5^th^ year students offering amongst others a simulation ward and a student-to-staff ratio of 6 on average could only be initiated due to tuition fees – and since its implementation it ranks among the courses evaluated best by the students [24]. That despite these measures that obviously aim at improving education the majority of students in our survey did not link tuition fees to enhanced quality of studies might be due to the fact that most of them simply do not know that tuition fees had been essential to make this possible and that most of them had not experienced their implementation. This latter interpretation is supported by our finding that particularly those students who never had to pay tuition fees refused them.

#### 4.4. Limitations of our study

The return rate in our online survey was only about 18%, though this is in an expected range regarding this kind of survey (e.g. [25]). Another limitation of our study is that our data are restricted to the University of Cologne. However, to our knowledge, similar data from other German universities have not been published (or even analyzed) yet and thus cannot be used for scientific purpose. Another shortcoming is that, compared to other countries, tuition fees in Germany have been rather low. Nonetheless, given that e.g. rents in Cologne rank among the highest students have to pay in Germany it is not surprising that our students had to spend additional time on earning money [26]. The more surprising is that time needed for their studies and dropout rates were not influenced.

## 5. Conclusion

Tuition fees did not affect discontinuation rate or study duration of Cologne medical students. However, fees obviously influenced the study course due to an increased need to pursue a sideline. Abrogation of these fees (or of the meanwhile introduced compensation payment by the federal state government) likely rules out many measures that aim at enhancing quality of medical studies (see above and for example [27]). To enhance students’ acceptance of tuition fees in the future, their use and (potential) benefit should be evaluated and communicated more intensively.

## Acknowledgements

We thank Mrs. Inka Meyer-Widynski for her help with data acquisition.

## Competing interests

The authors declare that they have no competing interests.

## Supplementary Material

Online survey

## Figures and Tables

**Table 1:**
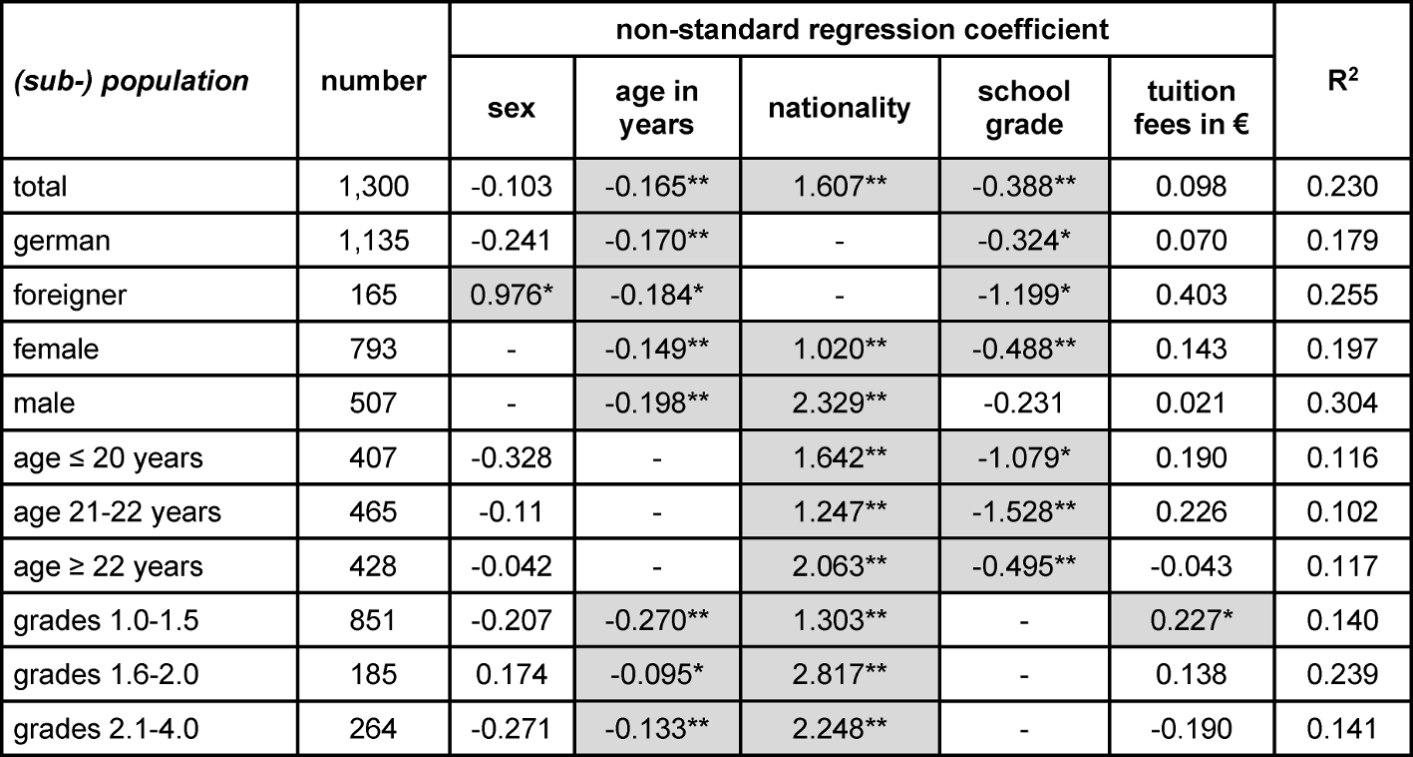
Results of a regression analysis regarding the fraction of students who kept standard study duration. *: p<0.05 (2-sided); **: p<0.01 (2-sided); coding: male=0, female = 1; foreigner = 0, German = 1; translation of the German school grades for “pass” in ECTS-grades: 1.0 to 1.5 = A (“excellent”); 1.6 to 2.0 = B (“good”), 2.1 to 3.0 = C (“satisfactory”), 3.1 to 4.0 = D/E (“sufficient”).

**Table 2:**
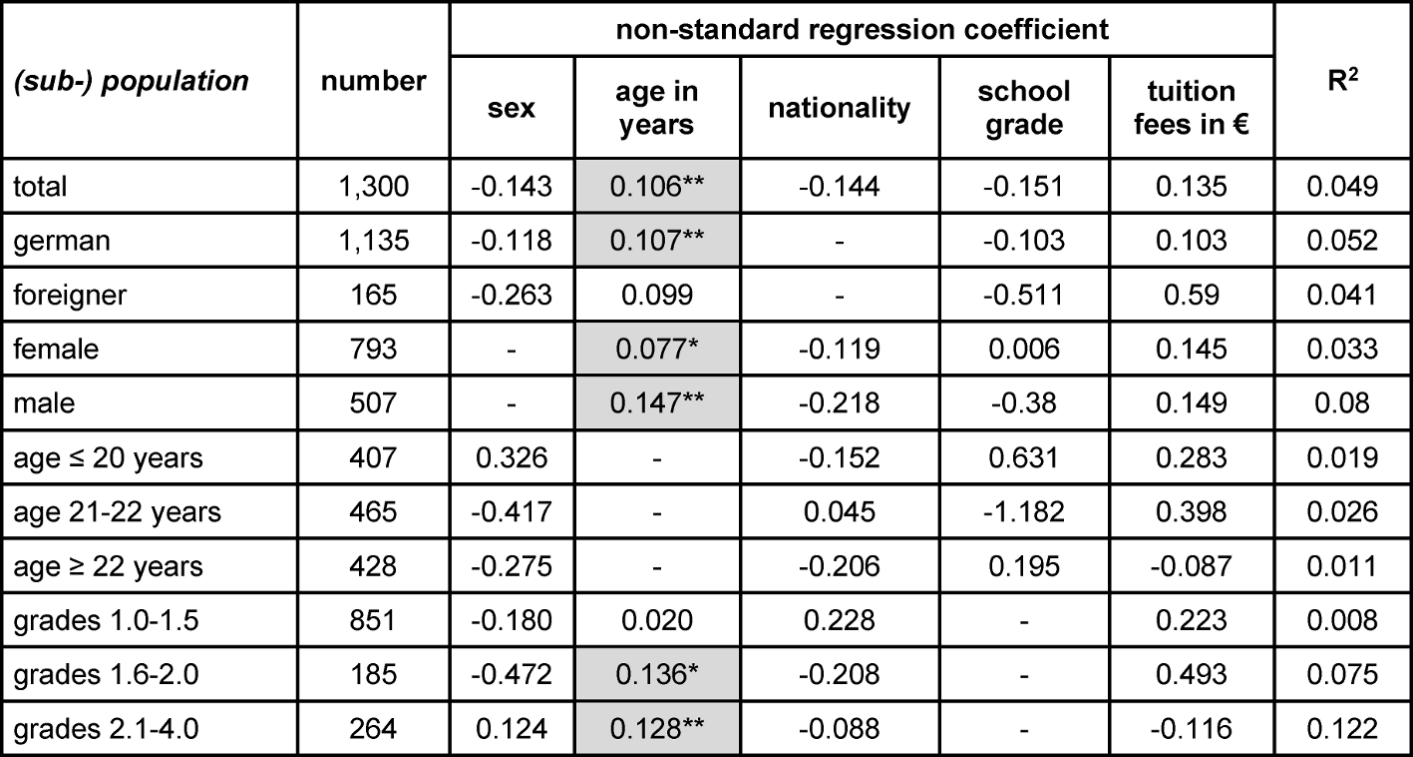
Results of a regression analysis regarding the fraction of students leaving university during the first two years of their studies. *: p<0.05 (2-sided); **: p<0.01 (2-sided); coding: male=0, female = 1; foreigner = 0, German = 1; translation of the German school grades for “pass” in ECTS-grades: 1.0 to 1.5 = A (“excellent”); 1.6 to 2.0 = B (“good”), 2.1 to 3.0 = C (“satisfactory”), 3.1 to 4.0 = D/E (“sufficient”).
