# Supplementary material for: A study on effects of and stance over tuition fees
Source: GMS J Med Educ. 2016 Feb 15;33(1):Doc6. doi: 10.3205/zma001005 (PMC4766930; doi:10.3205/zma001005)
Supplement: Online survey [file JME-33-6-s-001.pdf]

English translation of the online survey on tuition fees

Personal data

1. sex ◻ femal ◻ male ◻ not specified
2. age in years __________
3. nationality ◻ German ◻ other: ___________ ◻ not specified

Occupational qualification of your parents

4. Graduation:

◻ none

◻ apprenticeship

◻ master craftsman

◻ polytechnic degree

◻ university degree

◻ graduation (e.g. PhD, MD)

◻ not specified

Current term of your studies

5. Which is the current term of your studies?

(If you have already graduated or if you meanwhile have stopped studying please choose the appropriate option)

◻ 1. term

◻ 2. term

…

Pre-degree ("Physikum")

6. Did you already pass the Physikum?

◻ yes

◻ no

◻ not specified

7. What was your preclinical study duration until you attended the pre-degree examination (for the first time)?

□ 4 terms (minimum study period)

□ 5 terms

□ 6 terms

□ 7 terms

□ 8 terms

□ 9 terms

□ 10 terms

□ > 10 terms

□ not specified

[if >4 terms have been chosen under 7.:]

8. Why have you not been able to keep the minimum study duration of four terms?

□ earning money additionally to my studies

□ failed examinations

□ raising own children

□ care of relatives

□ illness

□ change of city where I study

□ secondary studies in addition to medicine

□ exam nerves

□ others: ________

□ not specified

Tuition fees

9. Did you have to pay tuition fees?

□ yes

□ no

□ not specified

[if "yes" was chosen under 9.:]

10. How have tuition fees been raised? (you can chose more than one item)

□ parents/family

□ stipend

□ (future) employer

□ loan

□ earning money

□ others: ________

□ keine Angabe

[if "yes" was chosen under 9.:]

Earning money

11. If you have earned money additionally to your studies: how many hours per week?

□ 1-10 hours

□ 11-20 hours

□ 21-30 hours

□ > 30 hours

[if "yes" was chosen under 9.:]

12. Did you continue earning money additionally to your studies after tuition fees had been abrogated? If "yes", how many hours per week?

□ 0 hours

□ 1-10 hours

□ 11-20 hours

□ 21-30 hours

□ > 30 hours

Personal view

13. I have preponed or delayed courses for at least one term, e.g. due to failed exams or intended.

□ totally disagree □ rather disagree □ neither nor □ rather agree □ totally agree □ not specified

14. In general tuition fees are justified.

□ totally disagree □ rather disagree □ neither nor □ rather agree □ totally agree □ not specified

15. I refuse reintroduction of tuition fees of 500 € per term.

□ totally disagree □ rather disagree □ neither nor □ rather agree □ totally agree □ not specified

16. Study conditions have improved due to tuition fees.

□ totally disagree □ rather disagree □ neither nor □ rather agree □ totally agree □ not specified

Own comments on tuition fees

17. Here you can place own comments on tuition fees:

____

Comments on this survey

18. Do you want to comment this survey? We are interested in your feedback!

Comment: _________________
